# Error Budget for Geolocation of Spectroradiometer Point Observations from an Unmanned Aircraft System

**DOI:** 10.3390/s18103465

**Published:** 2018-10-15

**Authors:** Deepak Gautam, Christopher Watson, Arko Lucieer, Zbyněk Malenovský

**Affiliations:** 1 Discipline of Geography and Spatial Sciences, School of Technology, Environments and Design, University of Tasmania, Hobart TAS 7005, Australia; Christopher.Watson@utas.edu.au (C.W.); Arko.Lucieer@utas.edu.au (A.L.); Zbynek.Malenovsky@utas.edu.au (Z.M.); 2 School of Agriculture, Food and Wine, Faculty of Sciences, University of Adelaide, Adelaide SA 5064, Australia; 3 Centre for Sustainable Ecosystem Solutions, School of Biological Sciences, University of Wollongong, Northfields Avenue, Wollongong NSW 2522, Australia; 4 Department of Remote Sensing, Global Change Research Institute CAS, Bělidla 986/4a, CZ-60300 Brno, Czech Republic

**Keywords:** aerial spectroscopy, UAS, UAV, spectroradiometer, footprint, geolocation, error propagation

## Abstract

We investigate footprint geolocation uncertainties of a spectroradiometer mounted on an unmanned aircraft system (UAS). Two microelectromechanical systems-based inertial measurement units (IMUs) and global navigation satellite system (GNSS) receivers were used to determine the footprint location and extent of the spectroradiometer. Errors originating from the on-board GNSS/IMU sensors were propagated through an aerial data georeferencing model, taking into account a range of values for the spectroradiometer field of view (FOV), integration time, UAS flight speed, above ground level (AGL) flying height, and IMU grade. The spectroradiometer under nominal operating conditions (8${}^{\circ }$ FOV, 10 m AGL height, 0.6 s integration time, and 3 m/s flying speed) resulted in footprint extent of 140 cm across-track and 320 cm along-track, and a geolocation uncertainty of 11 cm. Flying height and orientation measurement accuracy had the largest influence on the geolocation uncertainty, whereas the FOV, integration time, and flying speed had the biggest impact on the size of the footprint. Furthermore, with an increase in flying height, the rate of increase in geolocation uncertainty was found highest for a low-grade IMU. To increase the footprint geolocation accuracy, we recommend reducing flying height while increasing the FOV which compensates the footprint area loss and increases the signal strength. The disadvantage of a lower flying height and a larger FOV is a higher sensitivity of the footprint size to changing distance from the target. To assist in matching the footprint size to uncertainty ratio with an appropriate spatial scale, we list the expected ratio for a range of IMU grades, FOVs and AGL heights.

## 1. Introduction

Airborne and space-borne spectroscopy have proven to be powerful techniques for vegetation status monitoring through estimation of biophysical and biochemical variables [1,2,3,4,5]. Burkart et al. [6] demonstrated the use of a small spot-measuring spectroradiometer mounted on a unmanned aircraft system (UAS) offering new potential across numerous applications including upscaling, calibration and validation of airborne and satellite sensors (e.g., airborne sensor HyPlant and/or the FLORIS sensor on-board of a future ESA FLEX mission [7,8,9]), and near-range retrieval of photosynthetic chlorophyll fluorescence emissions [10,11,12,13]. To fully exploit this technology on a UAS platform [6,14,15,16], accurate geolocation of the spectroradiometer measurements is required. This study aims to propagate the input uncertainties originated from position and orientation sensors to analyse the footprint geolocation accuracy of spectroradiometer observations as a function of the sensor field of view (FOV), above ground level (AGL) height, grades of the inertial measurement unit (IMU), spectroradiometer integration time, and UAS flight speed.

Although propagation of input uncertainties through georeferencing models for UAS mounted active sensors, such as an airborne LiDAR [17,18,19], and passive imaging cameras or scanners [20,21,22,23] has been extensively studied, the UAS-based non-imaging spectroradiometer requires a variation to the existing georeferencing approach. The challenge arises from technical differences in georeferencing applicable to those sensors. The georeferencing technique for an imaging sensor (e.g., a camera, hyperspectral scanner or a spectral 2D imager) is guided by the acquired image and ground control points (GCPs), especially when using the tie points between multiple images, and the spatial relations between multiple pixels of an image. This information is, however, not available in the case of a non-imaging spectroradiometer observation, which integrates the signal within a predefined FOV to produce a single radiance measurement. Similarly, georeferencing of laser measurements (e.g., a LiDAR) is guided by time-stamped range measurements from the sensor to the target, which is not available for a single spot measuring spectroradiometer observation. This lack of a pixel array within a footprint, tie points between subsequent measurement samples or, distance measurements to the target makes this geolocation task unique and worthy of investigation [24].

We focus on geometrical aspects of the spectroradiometer footprint and do not address the FOV required to achieve the user-specific signal-to-noise ratio of spectral observations. In most cases, a circular FOV of the spectroradiometer projected on a distant plane defines the sensor footprint. Footprint geolocation can be estimated from position and orientation of the spectroradiometer using a modified direct georeferencing technique used for aerial laser scanning [25,26,27,28]. Any uncertainty in geolocation has the potential to result in radiance interpretation error for the estimated geolocation.

The uncertainty in the footprint geolocation arises primarily from the uncertainty associated with the position (originated from global navigation satellite system (GNSS)) and attitude (originated from IMU) of the spectroradiometer at the moment of spectral acquisition. Additionally, there is also uncertainty associated with the calibration of device geometry, including the lever-arm offset and boresight angle (further complicated if using an independently orientated gimbal system often used to maintain a near-nadir view of the spectroradiometer’s fore optics). Some of the uncertainties associated with the different components of the acquisition system are assumed stationary (i.e., white noise), whereas others exhibit a temporal correlation that is best quantified using a coloured noise model. The determination of uncertainty in the footprint geolocation, therefore, requires appropriate consideration of each source of uncertainty (magnitude and stochastic behaviour), and subsequent propagation through the georeferencing model.

In this study, we use a simulation to investigate the spatial uncertainty of the footprint geolocation of a spectroradiometer mounted on a gimbal of a UAS. We focus on the assessment of a nominal lightweight sensor deployable on-board small UAS (<15 kg). Sensor noise characteristics (stationary and time correlated) are investigated using lab-based and in-flight experiments. All identifiable uncertainties were quantified and propagated through a georeferencing model to yield the overall footprint geolocation uncertainty. Finally, to provide operational recommendations for non-imaging UAS spectroscopy, we assess the relative contributions to the overall footprint uncertainty originating from independent error sources as well as from variation in flight parameters (i.e., flying height, flight speed, FOV, grade of IMU/GNSS, and spectroradiometer integration time).

## 2. Materials and Methods

### 2.1. Scientific Sensors and Platform

**Figure 1:**
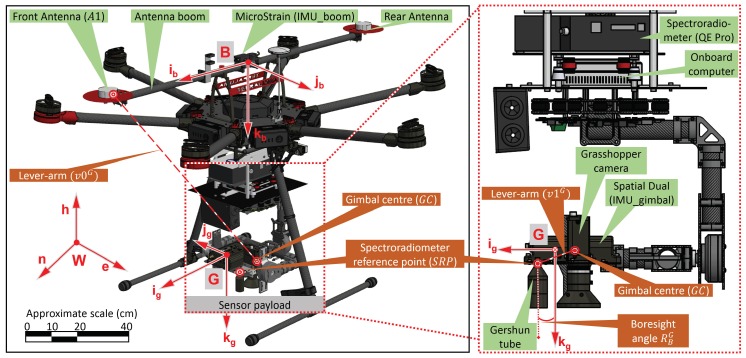
Detailed layout of the UAS spectroradiometer sensors (GNSS, IMUs, camera and spectroradiometer) mounted on a multirotor UAS.

A typical sensor payload for UAS spectroscopy includes a spectroradiometer, GNSS (to measure the sensor position), an IMU, and a camera. For this study, we consider a remotely controlled heavy-lift multirotor DJI Matrice 600 (Dà-Jiāng Innovations Science and Technology Co., Ltd., Shenzhen, China) equipped with custom-built antennae boom on the airframe and sensor package in the gimbal. The sensor package in the gimbal consisted of, a spectroradiometer (Ocean Optics QE Pro, Largo, FL, USA) with spectral sampling (FWHM 0.7 nm) within 500–850 nm wavelength, a MEMS-based IMU (Advanced Navigation Spatial Dual, Sydney, Australia) referred to here as IMU_gimbal, and a machine vision camera (FLIR Grasshopper GS3-U3-23S6M-C, Wilsonville, Oregon, USA). The antennae boom carried two dual frequency GNSS antennae connected to the IMU_gimbal (with Trimble BD982 GNSS receiver), a MEMS-based IMU (LORD MicroStrain 3DM-GX3-35, Williston, VT, USA) referred in this paper as IMU_boom to measure the orientation of the antennae boom, and an L1/CA GNSS antenna of the IMU_boom (see Figure 1).

The spectroradiometer’s downward-looking fibre optic cable records radiance of a target from nadir via an FOV restrictor called a Gershun tube which is rigidly mounted close to the IMU_gimbal and camera. The gimbal attempts to dampen the effect of flight dynamics and motor vibration on the Gershun tube. This is essential in UAS aerial spectroscopy in order to sustain a nadir pointing sensor configuration during spectral acquisitions, enabling a longer signal integration time over a target.

### 2.2. Sensor Geometry

The two GNSS antennae were mounted at a distance of 1.04 m (along the UAS flying direction), while the IMU_boom was positioned at the midpoint. This design was necessary to compute the lever-arm corrected position of the spectroradiometer reference point (SRP). Additionally, the antenna boom was approximately aligned with the IMU_gimbal by fixing the gimbal heading axis. The gimbal was therefore only used to attenuate the roll and pitch angle but not the heading such that the absolute heading computed using the dual antennae was a representative heading of the sensor payload (spectroradiometer, camera, IMU_boom, and IMU_gimbal). With the IMU mounted on the gimbal and the GNSS receiver mounted on the airframe, the data from the GNSS and IMU were acquired independent of each other [29]. This loosely-coupled configuration of the GNSS and IMU was adopted as the spectroradiometer which required a stable platform (via the gimbal) for data acquisition, whereas, the GNSS antenna required a clear sky view (mounted on top of the airframe).

### 2.3. Input Uncertainties

Small form-factor MEMS-based IMUs and dual frequency dual antennae GNSS employed to measure the pose of the UAS sensor payload are associated with multiple error sources such as: (i) synchronisation between different sensors, (ii) inherent noise in sensor measurements, and (iii) uncertainties in sensor calibration. The synchronisation error is regarded as negligible, as both sensors (IMUs and spectroradiometer) are synchronised by sensor-triggered pulses (with delay negligible compared to other error sources) in accordance with GNSS timestamps.

The inherent sensor noise includes GNSS position uncertainty (related to the coordinate frame expressed in the Geocentric Datum of Australia 1994 and projected using the Universal Transverse Mercator projection, grid zone 55, epsg: 28,355 [30], W=(n,e,h)) and IMU orientation uncertainty (about IMU_boom and IMU_gimbal coordinate frame defined by $B=(i_{b},j_{b},k_{b})$ and $G=(i_{g},j_{g},k_{g})$, respectively) (Figure 1). IMU orientation uncertainty is divided into IMU White Gaussian noise (WGN), IMU constant bias, time correlated IMU drift, and IMU turn-on to turn-on bias (defined as the variation in the constant bias of the IMU angles for each power cycle). Similarly, the calibration error is broken down into lever-arm uncertainty (about *G* coordinate frame) between the GNSS antenna and the spectroradiometer, and physical boresight uncertainty between the IMU and the spectroradiometer. The IMU constant bias and the constant physical boresight angle are, in this study, combined in a single constant boresight angle uncertainty. Similarly, the lever-arm uncertainty includes measurement uncertainties in multiple lever-arms, specifically from the front GNSS antenna (A1) to the gimbal centre (GC), and from GC to SRP (see Figure 1). The independent error sources used in this study to estimate the priori 3D uncertainty in footprint geolocation are outlined in Table 1.

We derived the standalone sensor measurement uncertainties and geometric calibration uncertainties based on the information provided by the sensor-specific technical specification in addition to custom experiments, and analysis of sensor data. Initially, seven sets of ground-based experiments were used to assess the achievable accuracy in position and orientation determination by comparing the IMU_gimbal with respect to a tactical-grade fibre optic gyroscope based GNSS/IMU. The MEMS-based GNSS/IMU, for a dynamic platform, was found to measure absolute position, and roll/pitch with accuracies better than 5 cm and 0.94${}^{\circ }$ respectively, for 95% of epochs [31]. While the position uncertainty derived from the experiment was used in this analysis, the orientation uncertainty required a further breakdown to contributing noise components including constant bias, turn-on to turn-on bias, WGN noise, and temporal drift. Thus, further experiments and analysis were performed on the IMU data to isolate these components.

The turn-on to turn-on bias uncertainty was modelled as a random process with zero mean and a variance derived from multiple power-cycle observations. Six sets of power cycle experiments were performed each with a length of 5–10 min and provision of sufficient cool-down time between each data collection. The IMU was kept in a fixed position during and in between the data collection stages by hard mounting it on a custom-built tribrach adaptor on a survey tripod. For each of the 6 stationary epochs, variation in averaged IMU orientation was used to estimate the turn-on to turn-on bias uncertainty. The effect of temporal drift on turn-on to turn-on bias was assumed to be negligible due to relatively short averaging time (1–2 min). Moreover, the WGN in the IMU is assumed to have an insignificant effect on the turn-on to turn-on bias determination due to averaging of high-frequency data (100 HZ) over the experiment duration. Turn-on to turn-on bias uncertainty by its nature is not feasible as it is impractical to calibrate the IMU after each power cycle; however, the experiment was designed to quantify its magnitude.

The bias of an IMU is known to drift slowly over time. This drifting bias may be described as a stochastic process that incorporates temporal correlation (i.e., coloured noise) driven by a random component. To identify and model the drifting bias present on the IMU, static data was collected for an extended period of time (2–13 h) at a 100 Hz frequency. During the entire experiment, the IMU was fixed on a custom-built tribrach adaptor on a survey tripod. The behaviour of the drift was assessed by performing Allan variance analysis. A first-order Markov recursive sequence was used to model the drift following Equation (1) [32,33,34].

(1) $$b_{d}\left(t\right)=sb_{d}\left(t-1\right)+w\left(t\right)$$

where $b_{d}\left(t\right)$ is the value of the drifting bias at an epoch *t*, *s* is an autocorrelation factor and w(t) is a zero-mean WGN with unknown variance $\sigma_{bd}^{2}$. During each iteration of the equation, the current value of the drifting bias ($b_{d}\left(t\right)$) is correlated with the previous value of the bias ($b_{d}\left(t-1\right)$) through the autocorrelation factor *s* and a sample from the WGN w(t) is added in to simulate the random component of the drifting bias. The autocorrelation parameter of the drift model was estimated by the temporal autocorrelation of the IMU measured attitude at a lag of a single epoch. The variance of the drifting bias was computed using the scale parameter and the variance of the IMU roll, pitch and heading angles ($\sigma_{\phi \theta \psi }^{2}$) as presented in Equation (2).

(2) $$\sigma_{bd}^{2}=\left(1-s^{2}\right)\sigma_{\phi \theta \psi }^{2}$$

As defined in Equation (1), drifting bias is time correlated. Traditional approaches to variance and covariance propagation are built under the assumption of stationary noise characteristics with a Gaussian distribution. Here, in order to proceed with error propagation for our purpose, we use a Gaussian noise magnitude defined by the 90% exceedance probability derived from simulations of the time correlated noise. The 90% exceedance was found to be higher (about 0.45${}^{\circ }$) in flying conditions as compared to the ground based or stationary case (about 0.25${}^{\circ }$) and were subsequently adopted for this study. To achieve this, the lab-based experiments contributed in defining the temporal drift parameters used to reconstruct the IMU temporal drift. The temporal drift parameters were then scaled to closely match the in-flight condition before determining the 90% exceedance temporal drift.

Similarly, the WGN noise present in the IMU was isolated by performing a moving average over the observed IMU orientation. This uncertainty was determined based on a static ground-based experiment and flight experiments. The WGN noise extracted from the IMU data in the static ground-based experiment also had a lower level of noise as compared to the flight experiment. The IMU WGN during flight was extracted by taking the orientation of the co-mounted camera (derived from bundle adjustment) as a reference.

The AGL height determination requires a digital surface model (DSM) of the study site, in addition to the spectroradiometer position. The DSM can be derived from a series of overlapping aerial images and surveyed GCPs. The achievable DSM accuracy depends on a number of variables including, but not limited to, the accuracy of GCPs, flying height, and spatial resolution of the image pixel. In this study, using literature as a guide [21,22,35,36], we assume the DSM uncertainty to be about twice as large as the uncertainty associated with a typical real-time kinematic GNSS surveying of the GCPs. Summing in quadrature, the uncertainty in the spectroradiometer position and DSM creation yields the total uncertainty in AGL height determination.

Additionally, there exists uncertainties associated with the sensor geometric calibration (lever-arm and boresight). The GNSS antenna was mounted at an offset to the SRP which created a lever-arm effect in measuring the position of the SRP. Similarly, the hard mounted IMU_gimbal and the spectroradiometer have angular misalignment in their mounting axes, creating a boresight angle. Thus, in situ measurements were required to determine the lever-arm offset and boresight angle. The boresight angle was estimated using a set of indoor controlled experiments by identifying and surveying the independent pointing angles of the hard-mounted sensors. To determine the lever-arm offset, measurement was required between the points SRP, A1, and GC. These points were, however, located inside the sensor housing body which made a mechanical measurement of the offsets impractical. Furthermore, before the measurement of the offset, any misalignment between the gimbal payload and the antenna boom needed to be resolved. Thus, we used a structure-from-motion (SfM) method which allowed both the estimation of the points (SRP, GC, and A1) as well as rotation to correct for misalignment, which collectively enabled a higher accuracy in offset measurement. For this, a 3D pointcloud of the UAS platform was generated using the photos of the airframe processed with SfM algorithm [37]. Surveyed control points were used to scale the 3D pointcloud. The offsets were measured within the scaled 3D pointcloud, after estimating the reference points and applying rotations, using AutoCAD software (Autodesk AutoCAD 2016) [38]. The surveying accuracy of the indoor experiments was used to estimate the uncertainty associated with the determination of the lever-arm offset and boresight angles.

#### IMU Error Modelling

**Figure 2:**
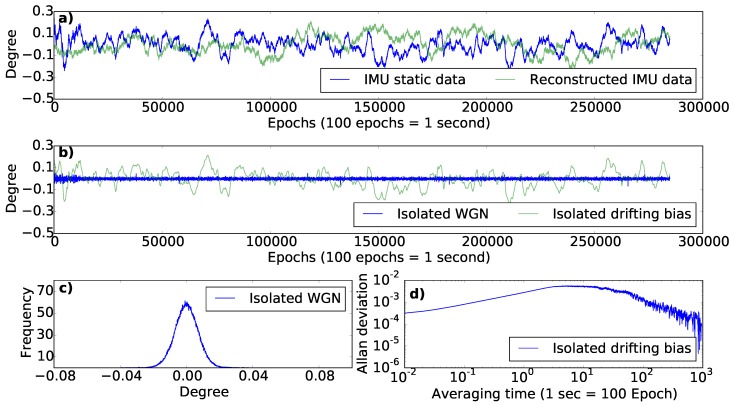
The IMU noise stochastic behaviour based on a static indoor experiment. (**a**) The static IMU data compared to the reconstructed modelled IMU data; (**b**) Decomposition of the static IMU data into drifting bias and WGN components; (**c**) The Gaussian distribution of the isolated WGN component; (**d**) The Allan deviation plot of the isolated drifting bias component.

The IMU_gimbal was assessed and modelled to estimate the drifting bias and WGN uncertainties associated with it (Figure 2). The static data of the IMU_gimbal (Figure 2a) constituted of WGN and drifting bias (Figure 2b), which were isolated by performing a moving average. The isolated WGN component of the noise had a Gaussian distribution (Figure 2c). Allan deviation analysis was performed on the isolated drifting bias for a range of averaging time. With the increase in averaging time, the effect of slow moving drift was seen to be more prevalent as marked by the rise in the Allan deviation (Figure 2d). The isolated drifting bias and WGN noise components were used to simulate the stationary IMU data, referred to hereon as reconstructed IMU data. The close match of the reconstructed IMU data with the actual data collected by the IMU provided verification of the IMU error estimates (Figure 2a).

The isolated standalone IMU uncertainties along with GNSS uncertainties, calibration and measurement uncertainties are summarised in Table 2. The calibration uncertainties (boresight and lever-arm) in Table 2 are estimated from a custom, indoor calibration experiment.

### 2.4. Error Propagation

The effect of error sources listed in Table 1 on the geolocation of the spectroradiometer footprint was determined by variance propagation of the input uncertainties through the direct georeferencing equation (Equation (3)). Geolocation of spectral measurements acquired with a spectroradiometer can be achieved photogrammetrically (using a camera as an additional sensor) or via a direct georeferencing approach using GNSS/IMU to measure position and orientation of the spectroradiometer. Given the extensive post-processing required for the photogrammetric approach of georeferencing [39], we focus here on the direct georeferencing approach. A spectroradiometer cannot measure the distance from its reference to the ground, therefore the range is derived as the height offset between the DSM to the location of the spectroradiometer (or AGL for the nadir pointing case). A modified direct georeferencing equation applicable for a UAS mounted spectroradiometer on a levelling gimbal is given by Equation (3) [25,40,41].

(3) $$\begin{array}{c} FP^{W}=A1^{W}+R_{B}^{W}R_{d}^{B}R_{b}^{B}R_{t}^{B}v0^{G}+R_{G}^{W}R_{d}^{G}R_{t}^{G}\left(v1^{G}+R_{b}^{G}v2^{G}\right) \end{array}$$

where $FP^{W}$ is the geolocation of the spectroradiometer footprint and $A1^{W}$ is the position of A1 both expressed in *W* coordinate frame. $R_{B}^{W}$ and $R_{G}^{W}$ are the attitude matrix as measured by the IMU_boom and IMU_gimbal (defining roll, pitch and heading from *W* to *B* and *G* coordinate system) respectively. The boresight misalignment between the IMU_boom and the antenna system is given by $R_{b}^{B}$ and similarly $R_{b}^{G}$ represents the boresight misalignment between the IMU_gimbal and the spectroradiometer. $R_{d}^{B}$ and $R_{d}^{G}$ represent the rotation matrix parametrised with the temporal drift component of the IMU_boom and IMU_gimbal, and $R_{t}^{B}$ and $R_{t}^{G}$ the respective turn-on to turn-on bias. The offset vector from A1 to the GC is given by $v0^{G}$. $v1^{G}$ represents the offset vector from the GC to the SRP, and $v2^{G}$ represents the offset vector from the SRP to the ground for the spectroradiometer pointing at nadir from a nominal AGL height. The input uncertainties variance and were propagated through the georeferencing equation (Equation (3)) to compute geolocation footprint uncertainty using Equation (4).

(4) $$C_{FP}=JC_{imput}J^{T}$$

where *J* (3×36) is the Jacobian matrix containing partial differential equations of the functional model (Equation (3)) with respect to each of the summarised 36 independent error sources in Table 1. $C_{input}$ (36×36) is the diagonal covariance matrix containing the input variances of each error source.

## 3. Results

**Figure 3:**
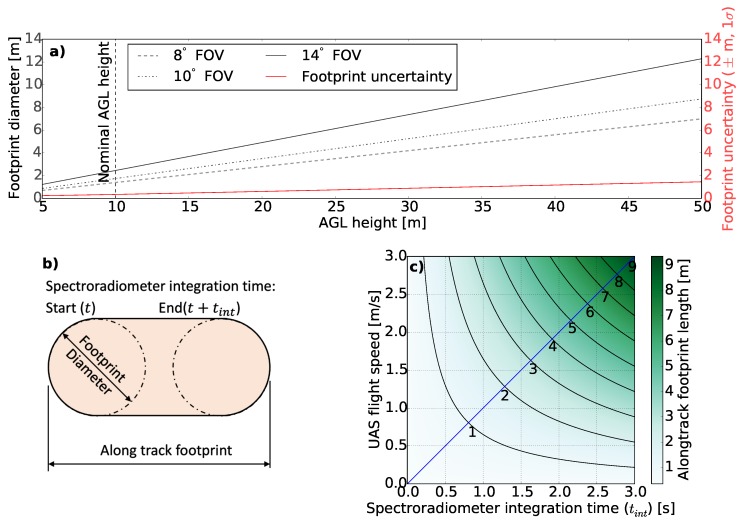
The shape and size of the UAS-mounted spectroradiometer footprint for a combination of: (**a**) AGL height and FOV; (**b**) spectroradiometer integration time $t_{int}$, and (**c**) UAS flight speed for a nominal AGL height of 10 m and FOV of 8${}^{\circ }$.

The shape and size of the spectroradiometer footprint were analysed for a range of FOVs, AGL heights, spectroradiometer integration times, and UAS flight speeds (Figure 3). The selection of FOV only influenced the size of the footprint, but had no impact on the accuracy of the footprint geolocation (Figure 3a). AGL height was found to be the prominent factor of both the size and geolocation uncertainty associated with the footprint (Figure 3a). For instance, for a constant FOV, a low flying height results in a smaller footprint and lower uncertainty compared to the footprint from a greater flying height. Thus in order to limit the uncertainty in footprint geolocation, it is optimal to fly lower and compensate the decrease in the size of the footprint by selecting a larger FOV. However, flying low requires a better ability to control the AGL distance in order to maintain a certain footprint size. In comparison, footprint size of a spectroradiometer at low AGL height is more susceptible to even smaller change in AGL distance.

Moreover, the combination of flight speed of the UAS and longer integration time of the spectroradiometer (typically 0.6 ms to 0.9 ms) results in elongation of the spectroradiometer footprint (Figure 3b). The diameter of the elongated circle is a function of FOV and AGL height whereas the along-track elongation of the footprint is a function of UAS flight speed and spectroradiometer integration time (Figure 3c). The UAS spectroradiometer system under investigation required an integration time of 0.6 s on a clear summer day in Hobart, Australia (−42.9${}^{\circ }$ S, 147.3${}^{\circ }$ E). This integration time combined with a flight speed of 3 m/s at a nominal AGL height of 10 m and FOV of 8${}^{\circ }$ resulted in a footprint diameter of 1.40 m and along-track footprint length of 3.20 m.

**Figure 4:**
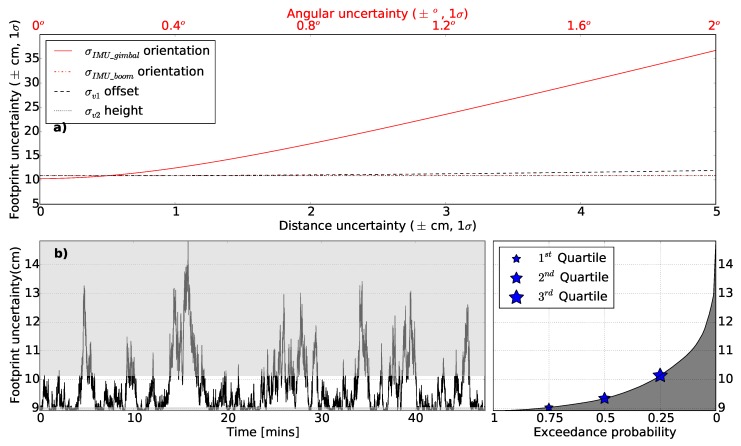
The footprint geolocation uncertainty for a range of: (**a**) dominant input uncertainties listed in Table 1 and (**b**) temporal drift. Most likely effect of the temporal drift (between exceedance probability 0.25 and 0.75) presented by the non-shaded region.

Among the listed input uncertainties in Table 2, the uncertainty associated with the IMU_gimbal was the second most sensitive parameter with the greatest impact on the total footprint error (Figure 4a). Other input uncertainties associated with the lever-arm offset, the boresight misalignment, the orientation of the IMU_boom, the AGL height, and the GNSS position had a smaller influence on the ground footprint uncertainty under the nominal condition. Furthermore, the drift present on the IMU_gimbal introduces temporally correlated uncertainty in the footprint geolocation estimation accounting for up to 5 cm additional uncertainty at a nominal AGL height (Figure 4b). The most likely effect of drift on footprint uncertainty (between exceedance probability 0.25 and 0.75) is presented by the non-shaded region in Figure 4b.

**Figure 5:**
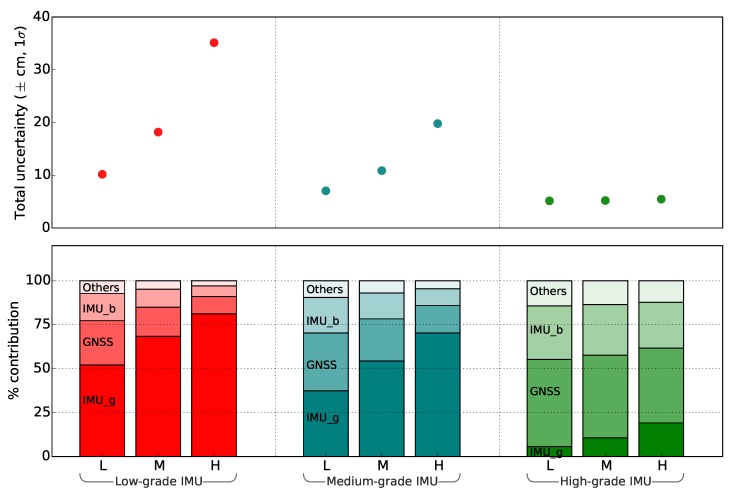
Comparative simulation of different grade IMUs (Low (equivalent to MicroStrain IMU), Nominal (equivalent to Spatial Dual IMU) and High (equivalent to a NovAtel’s SPAN CPT IMU)) at different AGL flying height (L = Low (5 m), M = Nominal (10 m), H = High (20 m)) to the footprint geolocation uncertainty. *NOTE: IMU_g and IMU_b represents IMU_gimbal and IMU_boom respectively.

Simulations were performed to assess the implication of a higher accuracy IMU (equivalent to NovAtel’s SPAN CPT IMU: see [42] for specification) used in the gimbal. Figure 5 shows a significant improvement in footprint geolocation accuracy, which nevertheless degraded with the increase in AGL height as expected. For example, for a much lower AGL height, a medium-grade (equivalent to Spatial Dual IMU: see [43] for specification) or lower-grade IMU (equivalent to MicroStrain IMU: see [44] for specification) achieved an accuracy comparable to that of a high-grade IMU operating at a much greater AGL height (Figure 5). However, using a lower grade IMU, the footprint uncertainty is expected to increase rapidly with increase in AGL height. Moreover, with the increased flying height, the contribution of IMU_gimbal towards the overall footprint error exceeded the contribution of other sensors and measurement errors. As expected, the rate of increase in footprint uncertainty with the AGL height was found highest using a low-grade IMU and lowest using a high-grade IMU (Figure 5). A low-grade IMU basically limits the ability to fly at a higher altitude and/or narrow down the FOV of the spectroradiometer.

For the UAS platform presented here, as per specifications (Table 2) flying at a nominal flying height (10 m), with nominal FOV (8${}^{\circ }$) the footprint uncertainty was computed to be approximately 11 cm, with a footprint uncertainty to size ratio of 0.08. A lower uncertainty to size ratio (see Table 3) indicates a better estimate of footprint geolocation in relation to the footprint size—this is particularly important when considering any post processing to account for spectral mixing over highly heterogeneous targets. A low-grade IMU limits our ability to reduce the FOV size without inflating the footprint to uncertainty ratio. For example, taking 0.1 as a desired ratio, a low grade IMU (at 10 m AGL height) cannot afford to utilise FOV lower than 14${}^{\circ }$, a high-grade IMU can employ all FOVs higher larger than 3${}^{\circ }$ whereas a nominal grade IMU cannot go lower than 8${}^{\circ }$ (Table 3). Incidentally, narrowing the FOV of our spectroradiometer system to lower than 8${}^{\circ }$ was limited by the amount of energy entering the spectroradiometer, specifically for retrieval of solar-induced chlorophyll fluorescence.

## 4. Discussion

We investigated the accuracy of spectroradiometer footprint geolocation for a range of sensor uncertainties (Table 2), flying parameters (spectroradiometer integration time, FOV, AGL height, and flight speed), and IMU grades (low, medium, and high-grade IMU). Our results indicate a footprint of approximately 1.4 m wide and 3.2 m long with an associated geolocation uncertainty of approximately 11 cm based on a medium-grade IMU, integration time of 0.6 s, flying height of 10 m, FOV of 8${}^{\circ }$, flight speed of 3 m/s, and taking into account the uncertainty sources as defined in Table 2. The estimated geolocation uncertainty can be considered as acceptable for homogeneous ground targets.

Flying height and orientation uncertainty of the IMU_gimbal were the primary factors influencing the spectroradiometer footprint geolocation uncertainty. Compared to medium and high-grade IMUs, the rate of increase in footprint uncertainty with the AGL height was found highest for a low-grade IMU. Our analysis under nominal flying height of 10 m and FOV of 8${}^{\circ }$ suggests that it is better to maintain or reduce the flying height and simultaneously increase the FOV in order to compensate the footprint area loss and increase signal strength. However, lowering the flying height is limited by the ability of the UAS to sustain a constant flying height above the terrain. Due to the low flying height and large FOV, the size of the footprint is more sensitive to subtle changes in the height above the terrain.

Considering a fixed flying height and FOV, the IMU orientation uncertainties were the main source of the footprint geolocation uncertainty, leading to a higher uncertainty to footprint size ratio. Using a high-grade IMU is particularly important if the objects under investigation are small and sparsely distributed. For example, when capturing spectral data of individual grape vine plants with individual plant canopy diameter of about 40–60 cm, a 3${}^{\circ }$ FOV spectroradiometer using a high-grade IMU at 5 m AGL height will result in a ratio of uncertainty to footprint size of 0.20. On the other hand, canopy level spectroscopy of medium-size trees (crown diameter over 3 m) can be performed using a low-grade IMU at 10 m AGL height and with 6${}^{\circ }$ FOV, which provides a ratio of uncertainty to footprint size of 0.17. In general, a lower grade IMU limits our ability to narrow down the sensor FOV and fly at a higher AGL height.

Apart from AGL height and IMU grade, the optimal size selection of FOV also requires careful consideration. To ensure low footprint uncertainty, with respect to the footprint size, it is recommended to set the FOV at least three times larger than the total possible uncertainty of the IMU, which is given by a combination of IMU WGN, boresight angle, drift, and turn-on to turn-on bias. For instance, if using an IMU with errors reaching as high as ±1${}^{\circ }$, then the spectroradiometer FOV is recommended to be no less than 3${}^{\circ }$. While a large FOV suits better to applications over homogenous targets where spatial resolution of the spectroradiometer is not that crucial, spatially heterogeneous or a small-size targets require a smaller FOV. As a general guide, the FOV is recommended to be as small as possible (depending on the application), however, not less than: 1) three times the total uncertainty from the IMU_gimbal, 2) the minimum required to achieve a certain application-specific ratio of footprint uncertainty to size, and 3) the minimum required to achieve a certain signal-to-noise ratio of acquired spectral observations.

A high footprint uncertainty to size ratio (Table 3) for heterogeneous or small-sized targets, can result in spectral contamination (due to misplacement of footprint), whose tolerance can vary per application. Computation of some robust broad-band vegetation indices, such as for instance NDVI, can tolerate more spectral contamination, whereas a weak vegetation signal such as chlorophyll fluorescence demands low contamination by non-vegetated surfaces as its retrieval requires narrow spectral bands and oxygen absorption features. Despite several attempts to use a spectroradiometer on a UAS to study vegetation spectral properties ([6,14,15,16,45]), the level of acceptable spectral contamination resulting from footprint uncertainty to size ratio is still not very well understood.

The signal strength recorded by the spectroradiometer is directly proportional to the size of the footprint and the spectroradiometer integration time. A smaller footprint, resulting from the combination of low AGL and small FOV, typically requires a longer integration time as compared to a larger footprint, which results from the combination of high AGL and large FOV. Increasing the integration time can produce a required signal-to-noise ratio despite a low FOV, however, it will be achieved at the expense of footprint shape elongation.

Compared to the static indoor experiment, we observed an increase of IMU WGN and drifting bias when computing the error budget with in-flight input uncertainties. This increase was likely due to a range of factors associated with flight dynamics, for example, increased platform vibration, small amounts of flexure within each lever arm, and uncertainty associated with the photogrammetric processing chain used as the reference. We conclude that in addition to a suitable sensor selection, the use of a gimbal to dampen the effect of vibrations is essential.

A methodological limitation of this study lies in the utilization of a linear error propagation model. The georeferencing Equation (3) was linearised using first order Taylor expansion under the nominal conditions of 10 m AGL height, nadir pointing sensor configuration, and sensor specifications presented in Table 2. Consequently, the approach can only guarantee accurate computation for conditions similar to the defined nominal acquisition conditions. Nonetheless, the actual flight data of our UAS showed that the attitude of the IMU_gimbal was well within the linear range of trigonometric functions (±5${}^{\circ }$: thus holding our assumption sin(angle)≈angle, and cos(angle)≈1).

Another limitation is the treatment of temporally correlated drifting bias. When propagating the variances, the 90% exceedance values of the temporally correlated drifting bias were used as Gaussian noise in the error propagation. We consider this value to be conservative for the purpose of footprint uncertainty assessment, but more rigorous determination of the temporal correlation of the footprint uncertainty was not considered of practical benefit for the UAS spectroradiometer system. Similarly, the input uncertainties were considered to be independent (i.e., covariance in Equation (4) was considered to be zero). However, there is a possibility of some correlation among the input error sources.

The proposed method for accurate footprint geolocation determination does not differentiate between spectrally unique or spectrally mixed responses within any given footprint. Accurate geolocation of the footprint aids interpretation of the spectral response depending on the relative size of the target and the sensor footprint. Understanding the accuracy of the footprint geolocation becomes increasingly critical for the appropriate interpretation over highly spatially heterogeneous targets. For example, with regards to the remote sensing retrieval of solar-induced chlorophyll fluorescence of vegetation canopies, the proposed methodology provides information on the geolocation of the retrieved fluorescence spectral emissions. This geolocation information, when combined with ancillary information (e.g., chlorophyll content of plants at the same location), can provide insight into the actual photosynthetic activity and fractional cover of vegetation within the footprint envelope. This can additionally help to spectrally isolate the vegetation chlorophyll fluorescence signal from the abiotic surrounding.

Figure 5 and Table 3 can serve as a guide for the uncertainty associated with a different combination of sensors. However, for an accurate replication of this error assessment, it is important to test the input uncertainty associated with each sensor device. The proposed approach could in the future be extended by incorporation of errors associated with the payload vibration, and their implication on geolocation uncertainty. Furthermore, advanced algorithms, such as simultaneous localization and mapping or Kalman filtering, could be incorporated to reduce the uncertainty associated with the on-board GNSS/IMU.

UAS-based spectroscopy represents a new technology that can facilitate new remote sensing applications, such as vegetation biomass estimation and stress assessments in precision agriculture [10], as well as upscaling and performance validation of optical signals and products recorded by high-altitude air- and space-borne optical sensors, e.g., the airborne hyperspectral spectroradiometer HyPlant [8] or the high spatial resolution multispectral sensors of the WorldView satellite series [46]. Nevertheless, the UAS technology has still limitations when studying spatially heterogeneous targets composed of small objects. To overcome this shortcoming, it requires to yield a strong spectral SNR from a small footprint on the Earth surface, which assumes use of a spectroradiometer with a smaller FOV and a short signal integration time coupled with a high-grade IMU. Unfortunately, current high-grade IMU modules are too heavy to satisfy the weight limits of UAS payloads. Smaller FOV and shorter integration times are still producing insufficient spectral SNR and slowing down the flying speed for the reduction of footprint elongation, which is constrained by achievable duration of a flight mission. To fulfil the above-mentioned application requirements, we need UAS technology of a higher payload capacity that can carry spectroradiometers with a fore optic capable to produce sufficiently large SNR, even when using narrow FOV and short integration times, and MEMS-based IMU technology providing sufficiently accurate sensor orientation data. This progression would collectively allow the UAS-based point spectrometry to work with small-size footprints collected from greater flying heights with acceptably low geolocation uncertainty, which opens new capabilities in observing even single plants over relatively large study areas.

## 5. Conclusions

We investigated the uncertainty in geolocation of the footprint for a spectroradiometer on-board a small unmanned aircraft system. The direct georeferencing technique applied in this study uses a global navigation satellite system receiver for sensor positioning and an inertial measurement unit for its orientation determination. We demonstrated how the variance of individual errors, originating from independent sources, can be estimated and propagated to estimate the overall accuracy of the spectroradiometer footprint geolocation. We assessed the relative error contribution of several input parameters such as the above ground level flying height, grade of IMU, spectroradiometer integration time, spectroradiometer field of view, and UAS flight speed in relation to the shape, size, and geolocation uncertainty of the footprint. The spectroradiometer footprint typically has the shape of an elongated circle, where the across-track width is determined by the FOV and AGL height, and the along-track length is the function of spectroradiometer integration time and UAS flight speed. The investigated UAS spectroradiometer footprint was 140 cm wide across-track and the 320 cm long along-track for the FOV of 8${}^{\circ }$, AGL height of 10 m, spectroradiometer integration time of 0.6 s, and UAS flight speed of 3 m/s. The geolocation uncertainty of this particular footprint was found to be ≈11 cm (footprint uncertainty to diameter ratio equal to 0.08).

The AGL flying height was found to be one of the major determinants of footprint accuracy for a given accuracy of the used IMU. When the flying height was fixed, the uncertainty in IMU measured orientation of the spectroradiometer had the second largest influence on the overall geolocation accuracy. With the increase in flying height, the rate of increase in footprint uncertainty was found to be higher for the lower grade IMU. Other factors, such as lever-arm offset uncertainty and orientation of the IMU on the airframe, had a relatively low impact. From a footprint error budget perspective, it is recommended to reduce the flying height and to increase the sensor FOV. A larger FOV will keep the footprint size comparable to those for higher AGL altitudes and simultaneously strengthen the signal recorded by a spectroradiometer. However, a lower AGL and larger FOV require accurate control of the AGL distance during flight operations. With the current pace of technological progress, we foresee development of new, more sensitive spectroradiometers that will allow for reduction of integration time, facilitating a narrower FOV, and resulting in small-size footprints. This will require a deep understanding of the spatial uncertainty in footprint location and extent as outlined and demonstrated in this work.

## Figures and Tables

**Table 1:** The input error components (originating from each sensor measurement and calibration) used for variance propagation to determine the footprint geolocation uncertainty of a UAS mounted gimballed spectroradiometer.

Sensor/Metric	Description	Symbolic Representation
GNSS antenna	3 uncertainties in the position	($\sigma_{n^{W}}$, $\sigma_{e^{W}}$, $\sigma_{h^{W}}$)
IMU_boom	3 WGN uncertainties in orientation	($\sigma_{r^{B}}$, $\sigma_{p^{B}}$, $\sigma_{h^{B}}$)
	3 boresight uncertainties	($\sigma_{r_{b}^{B}}$, $\sigma_{p_{b}^{B}}$, $\sigma_{h_{b}^{B}}$)
	3 drift uncertainties in orientation	($\sigma_{r_{d}^{B}}$, $\sigma_{p_{d}^{B}}$, $\sigma_{h_{d}^{B}}$)
	3 turn-on to turn-on bias uncertainties	($\sigma_{r_{t}^{B}}$, $\sigma_{p_{t}^{B}}$, $\sigma_{h_{t}^{B}}$)
IMU_gimbal	3 WGN uncertainties in orientation	($\sigma_{r^{G}}$, $\sigma_{p^{G}}$, $\sigma_{h^{G}}$)
	3 boresight uncertainties	($\sigma_{r_{b}^{G}}$, $\sigma_{p_{b}^{G}}$, $\sigma_{h_{b}^{G}}$)
	3 drift uncertainties in orientation	($\sigma_{r_{d}^{G}}$, $\sigma_{p_{d}^{G}}$, $\sigma_{h_{d}^{G}}$)
	3 turn-on to turn-on bias uncertainties	($\sigma_{r_{t}^{G}}$, $\sigma_{p_{t}^{G}}$, $\sigma_{h_{t}^{G}}$)
Lever-arm	3 offset uncertainties from A1 to GC	($\sigma_{v0_{i}^{G}}$, $\sigma_{v0_{j}^{G}}$, $\sigma_{v0_{k}^{G}}$)
	3 offset uncertainties from GC to SRP	($\sigma_{v1_{i}^{G}}$, $\sigma_{v1_{j}^{G}}$, $\sigma_{v1_{k^{G}}}$)
AGL	3 offset uncertainties from SRP to nadir	($\sigma_{v2_{i}^{G}}$, $\sigma_{v2_{j}^{G}}$, $\sigma_{v2_{k}^{G}}$)

Note on symbolic representation: Term $\sigma_{x_{y}^{Z}}$ represents; uncertainty of the term $x_{y}$ in *Z* coordinate frame.

**Table 2:** The estimated standalone uncertainties in sensor measurement and calibration.

Sensor/Metric	Uncertainties	Nominal Value (±1σ) [cm or deg]	Method of Determination
GNSS antenna	($\sigma_{n^{W}}$, $\sigma_{e^{W}}$, $\sigma_{h^{W}}$)	(3.0, 3.0, 4.0)	Ground based experiments
	($\sigma_{r^{B}}$, $\sigma_{p^{B}}$, $\sigma_{h^{B}}$)	(0.4, 0.4, 0.9)	User manual and static data
	($\sigma_{r_{b}^{B}}$, $\sigma_{p_{b}^{B}}$, $\sigma_{h_{b}^{B}}$)	(0.2, 0.2, 0.2)	Assumed (value is not critical)
	($\sigma_{r_{d}^{B}}$, $\sigma_{p_{d}^{B}}$, $\sigma_{h_{d}^{B}}$)	(0.45, 0.45, 0.9)	Projected for flight condition.
IMU_boom	($\sigma_{r_{t}^{B}}$, $\sigma_{p_{t}^{B}}$, $\sigma_{h_{t}^{B}}$)	(0.10, 0.12, 0.58)	Power cycle experiments
	($\sigma_{r^{G}}$, $\sigma_{p^{G}}$, $\sigma_{h^{G}}$)	(0.2, 0.2, 0.1)	User manual, dynamic and static data
	($\sigma_{r_{b}^{G}}$, $\sigma_{p_{b}^{G}}$, $\sigma_{h_{b}^{G}}$)	(0.2, 0.2 0.2)	Approximated from calibration Experiments
	($\sigma_{r_{d}^{G}}$, $\sigma_{p_{d}^{G}}$, $\sigma_{h_{d}^{G}}$)	(0.25, 0.25, 0.55)	90% exceedance of reconstructed temporal drift
	($\sigma_{r_{t}^{G}}$, $\sigma_{p_{t}^{G}}$, $\sigma_{h_{t}^{G}}$)	(0.08, 0.08, 0.45)	Power cycle experiments
IMU_gimbal	($\sigma_{v0_{i}^{G}}$, $\sigma_{v0_{j}^{G}}$, $\sigma_{v0_{k}^{G}}$)	(0.5, 0.5, 0.5)	Measured from 3D point cloud
Lever-arm	($\sigma_{v1_{i}^{G}}$, $\sigma_{v1_{j}^{G}}$, $\sigma_{v1_{k^{G}}}$)	(0.5, 0.5, 0.5)	Measured from 3D point cloud
AGL	($\sigma_{v2_{i}^{G}}$, $\sigma_{v2_{j}^{G}}$, $\sigma_{v2_{k}^{G}}$)	(5.0, 5.0, 7.0)	Approximated from $\sigma_{h^{W}}$

**Table 3:** The ratio of footprint diameter and footprint geolocation uncertainty using nominal grade sensors (Table 2) at a nominal AGL height of 10 m (and 5 m) for a range of FOV achievable using Ocean Optics Gershun tube kit.

FOV	Footprint Diameter (cm)	Ratio of Footprint Uncertainty (±1σ) to Size		
		L-G IMU	M-G IMU	H-G IMU
1${}^{\circ }$	17.5 (8.7)	1.04 (1.17)	0.62 (0.81)	0.30 (0.59)
2${}^{\circ }$	34.9 (17.5)	0.52 (0.58)	0.31 (0.40)	*0.15* (0.29)
3${}^{\circ }$	52.4 (26.2)	0.35 (0.39)	0.21 (0.27)	*0.10* (0.20)
6${}^{\circ }$	104.8 (52.4)	*0.17 (0.19)*	*0.10 (0.13)*	**0.05** *(0.10)*
8${}^{\circ }$	139.9 (70.0)	*0.13 (0.15)*	**0.08** *(0.10)*	**0.04 (0.07)**
10${}^{\circ }$	175.0 (87.5)	*0.10 (0.12)*	**0.06 (0.08)**	**0.03 (0.06)**
14${}^{\circ }$	245.6 (122.8)	**0.07 (0.08)**	**0.04 (0.06)**	**0.02 (0.04)**
16${}^{\circ }$	281.1 (140.5)	**0.06 (0.07)**	**0.04 (0.05)**	**0.02 (0.04)**
20${}^{\circ }$	352.7 (176.3)	**0.05 (0.06)**	**0.03 (0.04)**	**0.01 (0.03)**
28${}^{\circ }$	498.7 (249.3)	**0.04 (0.04)**	**0.02 (0.02)**	**0.01 (0.02)**

L-G = Low-grade, M-G = Medium-grade, H-G = High-grade; Nominal category of footprint uncertainty to size ratio (Large, *Medium*, and **Small**); Numerical values in parentheses are computed for 5 m AGL height.
